# Efficacy and Safety of Novel Agent-Based Therapies for Multiple Myeloma: A Meta-Analysis

**DOI:** 10.1155/2016/6848902

**Published:** 2016-02-01

**Authors:** Xiaoxue Wang, Yan Li, Xiaojing Yan

**Affiliations:** Department of Hematology, The First Hospital, China Medical University, Shenyang 110001, China

## Abstract

This study aimed at comparing bortezomib, thalidomide, and lenalidomide in patients with multiple myeloma (MM) for safety and efficacy using meta-analysis. This meta-analysis identified 17 randomized controlled trials (RCTs) including 6742 patients. These RCTs were separated according to the different agent-based regimens and to autologous stem-cell transplantation (ASCT). Complete response (CR), progression-free survival (PFS), overall survival (OS), and adverse events (AE) were combined. The total weighted risk ratio (RR) of CR was 3.29 [95% confidence interval (95% CI): 2.22–4.88] (*P* < 0.0001) for the novel agent-based regimens. These novel agent-based regimens showed greater benefit in terms of PFS of all subgroups irrespective of whether the patient received ASCT or not. The hazard ratio (HR) for PFS was 0.64 [95% CI: 0.60–0.69] (*P* < 0.00001). Improvements of OS could be found only in the bortezomib- and thalidomide-based regimens without ASCT. The pooled HRs were 0.74 [95% CI: 0.65–0.86] (*P* < 0.0001) and 0.80 [95% CI: 0.70–0.90] (*P* = 0.0004), respectively. Several AEs were shown more frequently in the novel agent-based regimens compared with controls such as hematologic events (neutropenia, anemia, and thrombocytopenia), gastrointestinal infection, peripheral neuropathy, thrombosis, and embolism events. In conclusion, in spite of the AEs, novel agent-based regimens are safe and effective for the treatment of MM.

## 1. Introduction

Multiple myeloma (MM) is a relatively common hematological malignancy characterized by the proliferative disorder of plasma cells in the bone marrow with excessive monoclonal protein production [1]. Median age at presentation is 66 years [2]. Age-adjusted incidence is 7 per 100,000 men and 4.6 per 100,000 women in the USA [3]. Risk factors for MM are ill defined, but likely risk factors are monoclonal gammopathy of undetermined significance, obesity, black race, and age [4, 5]. Median survival for newly diagnosed MM is about 44.8 months [6]. MM cannot be cured [1], but new drugs are available to manage patients with MM.

Indeed, over the last decade, many randomized clinical trials (RCTs) have been undertaken to demonstrate that novel agents such as thalidomide, lenalidomide, and bortezomib as induction/consolidation/maintenance treatments have a clear superiority for improving the outcomes of patients with MM, therefore leading to high rates of response and improved progression-free survival (PFS) and overall survival (OS), irrespective of whether the patient received autologous stem-cell transplantation (ASCT) or not. Indeed, it has been shown that patients with MM treated with thalidomide, lenalidomide, or bortezomib had a median survival of 30.9 months compared with 14.8 months for patients who did not receive these drugs [6]. However, there is a lack of studies reviewing these RCTs in terms of meta-analysis.

Therefore, the present study aimed at comparing the safety and efficacy of bortezomib, thalidomide, and lenalidomide in patients with MM using meta-analysis.

## 2. Materials and Methods

### 2.1. Retrieval Strategy

PubMed/Medline, Embase, Science Direct, OVID, Cochrane Controlled Trials Register, International Standard Randomized Controlled Trial Number, and https://www.clinicaltrials.gov/ were searched for RCTs using the medical subject headings (“multiple myeloma” [Title]) AND (bortezomib [Title] OR thalidomide [Title] OR lenalidomide [Title]), species = human, and published between April 2005 and April 2015. Additional relevant trials and practice guidelines were hand-searched according to the reference lists of the identified articles (all data were updated to April 2015).

### 2.2. Selection Criteria

Inclusion criteria were as follows: (1) prospective phase III RCT was performed in patients with MM; (2) the intervention used novel agent-based regimens like bortezomib, thalidomide, or lenalidomide; (3) the controls received conventional treatments or placebo; (4) the article must provide sufficient information to calculate the risk ratio (RR) for complete response (CR) and crude hazard ratios (HRs) for PFS and OS; (5) adverse effects (AEs) were provided; (6) the article was published in English; and (7) the full text was available.

Exclusion criteria were as follows: (1) retrospective study or non-RCT; (2) study not focusing on the treatment of MM; (3) study not providing survival data such as HR, RR, or survival curves; or (4) letters, meeting proceedings, reviews, or abstracts.

Multiple reports about a single study were considered as one publication, and the final updated data was included in the present analysis. If specific data were not reported in the final report, they were extracted from a preceding report.

### 2.3. Quality Assessment and Control

All the titles and abstracts of retrieved articles were independently reviewed by two investigators (W. X. X. and Y. X. J.) for the inclusion/exclusion criteria. Any divergent opinions were resolved through discussion. The quality of the trials was evaluated using the Jadad quality scores [7] including methods for randomization, generation of allocation concealment, blinding, follow-up, description of dropouts, and intention-to-treat (ITT) analyses.

### 2.4. Collection of Data

The primary outcomes of the present meta-analysis were complete response (CR), progression-free survival (PFS), and overall survival (OS). The secondary outcome was AEs. Treatment response and disease progression were reported by investigators according to the criteria of the European Group for Blood and Marrow Transplantation (EBMT) [8]. OS was measured from the date of enrollment, randomization, or start of treatment until death from any cause. The grades of AEs were assessed using the National Cancer Institute Common Terminology Criteria for Adverse Events (NCI-CTCAE), version 3.0. The trial number, authors, years of publication, country of investigators, sample size, treatment regimens, follow-up, curative effects, and AEs of each RCT were extracted. Data extraction was independently made by the two investigators (W. X. X. and Y. X. J.).

### 2.5. Statistical Analysis

All meta-analyses were completed using REVMAN version 5.2. Between-study and between-subgroup heterogeneity were tested using the Cochrane chi-square test and quantified using the *I* ^2^-statistic. When *I* ^2^ > 50%, we considered that there was heterogeneity and selected the random effect model. When *I* ^2^ ≤ 50%, we considered that there was no heterogeneity and selected the fixed effect model. Dichotomous data (CR) were expressed as RR using a 95% confidence interval (CI). Time-to-event data (PFS and OS) were pooled and reported as hazard ratio (HR). Forest plots of HRs were completed using the Exp[(*O* − *E*/*V*)] method. Events and total number of participants in novel agent-based regimens and control arms were also entered. The concrete HR and 95% CI were directly used if they were available in the literature. If not, Engauge Digitizer V4.1 was used to estimate the survival rates at any point on the survival curves. Then, the variance and *O* − *E* were calculated using the method by Tierney et al. [9]. Funnel plot analysis concerning potential publication bias was also performed to confirm the publication bias. *P* < 0.05 indicated statistical significance.

## 3. Results

### 3.1. Description of Trials

**Figure 1:**
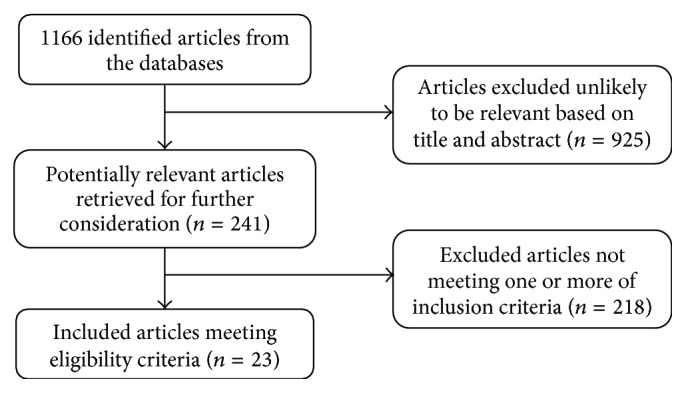
Selection procedure of studies.

A comprehensive literature search was performed. The initial search yielded 1166 articles, of which 23 articles (17 RCTs) were finally included in the present meta-analysis [10–30] (Figure 1). These RCTs included 6742 patients. These RCTs included five RCTs that tested bortezomib-based regimens (including four which involved ASCT), ten RCTs that tested thalidomide-based regimens (including two which involved ASCT), and two RCTs that tested lenalidomide-based regimens (both without ASCT). All RCTs were reported as full articles. All studies reported intention-to-treat (ITT) analyses and description of dropouts except for one. Four trials were double-blinded. The characteristics of the included trials are described in Table 1.

### 3.2. Complete Response

**Figure 2:**
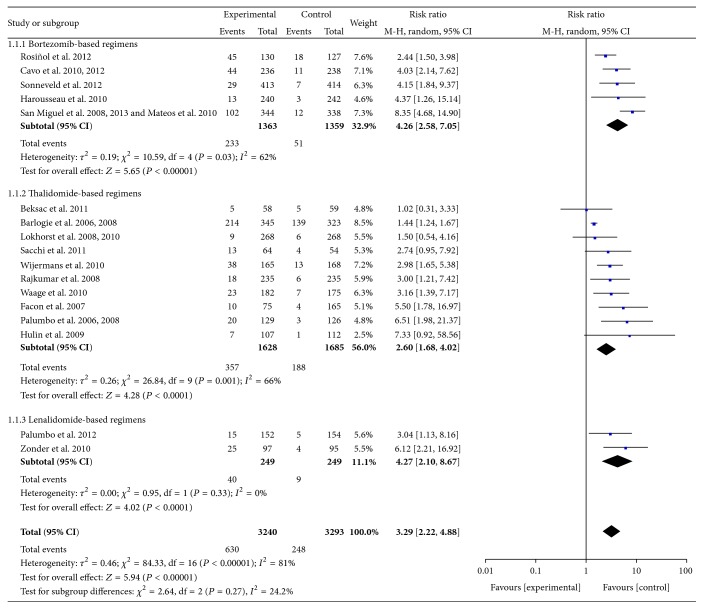
Meta-analysis of complete response rate with novel agent-based regimens.

Figure 2 illustrates a meta-analysis of the response effect from all RCTs using novel agent-based regimens. The CR rate of patients with MM was consistently improved by the novel agent-based regimens compared with controls. The weighted RRs of CR were 4.26 [95% CI 2.58–7.05] for bortezomib-based regimens, 2.60 [95% CI 1.68–4.02] for thalidomide-based regimens, and 4.27 [95% CI 2.10–8.67] for lenalidomide-based regimens (*P* < 0.001 in all three subgroups). The overall weighted RR of CR was 3.29 [95% CI 2.22–4.88; *P* < 0.0001]. Heterogeneity could be found among the trials with bortezomib and thalidomide RCTs (*P* = 0.03 and *P* = 0.001, resp.), but not in the lenalidomide RCTs. Test for subgroup differences was negative (*P* = 0.27). There was no significant difference between subgroups when comparing the groups between novel agent-based regimens with and without ASCT (*P* = 0.18, *I* ^2^ = 45.1%) (Table 2).

### 3.3. Progression-Free Survival

**Figure 3:**
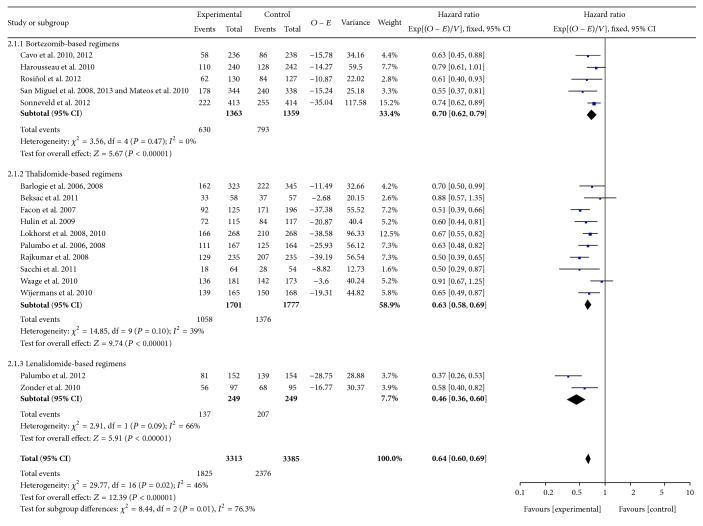
Meta-analysis of progression-free survival with novel agent-based regimens.

Figure 3 illustrates a meta-analysis of PFS data among bortezomib-, thalidomide-, and lenalidomide-based trials with or without ASCT. The pooled HRs for PFS were 0.55 [95% CI 0.37–0.81] (*P* = 0.002) for bortezomib-based regimens without ASCT and 0.72 [95% CI 0.64–0.82] (*P* < 0.00001) for bortezomib with ASCT. HRs were 0.62 [95% CI 0.55–0.69] (*P* < 0.00001) and 0.68 [95% CI 0.57–0.81] (*P* < 0.0001) when comparing thalidomide-based therapy with or without ASCT with controls, respectively. As for the lenalidomide-based regimens without ASCT, the HR was 0.46 [95% CI 0.36–0.60] (*P* < 0.00001). However, there were differences when comparing the groups between novel agent-based regimens with and without ASCT (*P* = 0.01, *I* ^2^ = 84.6%) (Table 2).

### 3.4. Overall Survival

**Figure 4:**
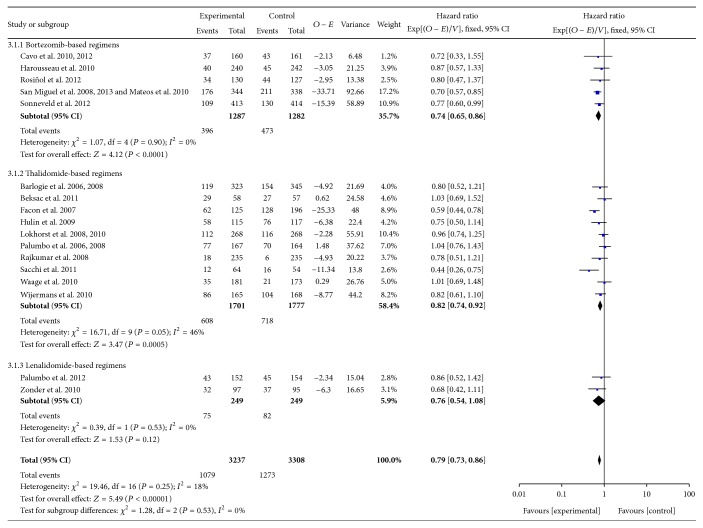
Meta-analysis of overall survival with novel agent-based regimens.

As shown in Figure 4, the pooled HRs for OS were 0.79 [95% CI 0.65–0.96] (*P* = 0.02) and 0.70 [95% CI 0.57–0.85] (*P* = 0.0005) for bortezomib-based regimens with or without ASCT, respectively, which suggested that bortezomib-based regimens could improve OS. In the subgroup of thalidomide-based regimens, the pooled HRs for OS were 0.91 [95% CI 0.73–1.14] (*P* = 0.41) and 0.80 [95% CI 0.70–0.90] (*P* = 0.0004) for therapy with or without ASCT, respectively. OS was not significantly improved by thalidomide-based regimens with ASCT. In addition, there was no clear advantage on OS in the lenalidomide-based regimens without ASCT. The pooled HR for OS was 0.76 [95% CI 0.54–1.08] (*P* = 0.12). There was no superiority of ASCT (*P* = 0.30, *I* ^2^ = 5.5%) (Table 2).

### 3.5. Adverse Events

In several studies included in this meta-analysis, data about Grades III/IV AEs were provided. Some frequently mentioned AEs such as hematologic events (neutropenia, anemia, and thrombocytopenia), gastrointestinal infection (GI), peripheral neuropathy, and thrombosis or embolism events were extracted among eligible studies.

**Figure 5:**
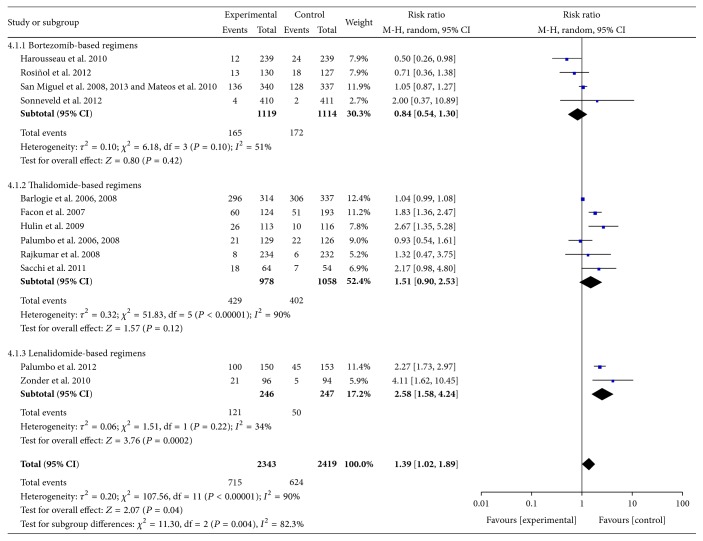
Comparison of novel agent-based regimens versus controls for neutropenia (Grades III-IV).

*(1) Neutropenia.* Data were available from 12 RCTs [10–13, 16–19, 21–23, 25, 26, 30–32]. These studies included 4762 patients. The pooled results showed statistically significant increases in the frequency of Grades III-IV neutropenia with the use of novel agent-based regimens compared with controls, especially in the lenalidomide-based group. The pooled RRs for neutropenia were 2.58 (95% CI 1.58–4.24; *P* = 0.0002) for the lenalidomide-based regimens and 1.39 (95% CI 1.02–1.89; *P* = 0.04) for all RCTs. The test for subgroup differences was positive (*P* = 0.004) (Figure 5).

**Figure 6:**
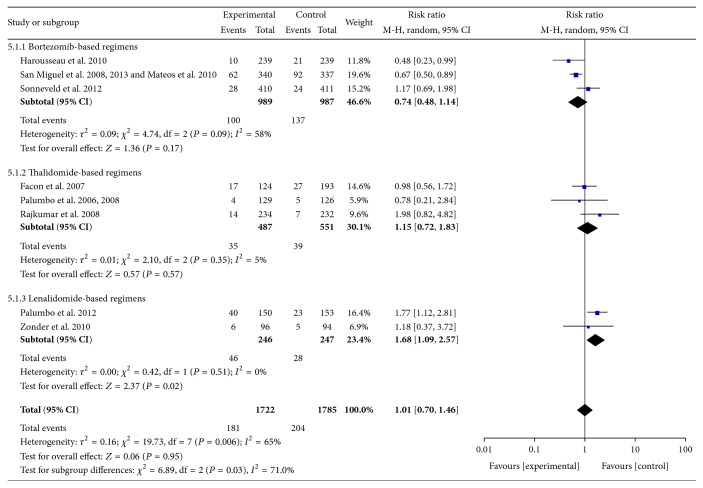
Comparison of novel agent-based regimens versus controls for anemia (Grades III-IV).

*(2) Anemia.* Data were available from 7 RCTs [10–13, 17, 18, 21–23, 31, 32]. These studies included 3507 patients. The pooled results showed significant increases in the frequency of Grades III-IV anemia with the use of lenalidomide-based regimens compared with controls. The pooled RR for anemia was 1.68 (95% CI 1.09–2.57; *P* = 0.02). There was heterogeneity among included RCTs (*I* ^2^ = 71%; *P* = 0.03) (Figure 6).

**Figure 7:**
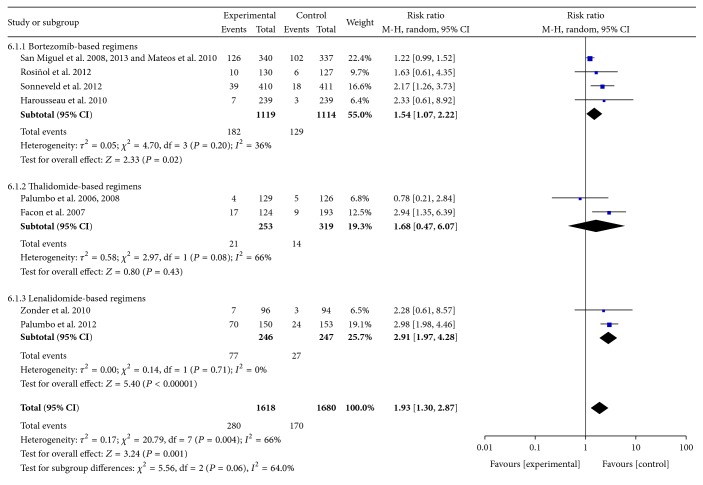
Comparison of novel agent-based regimens versus controls for thrombocytopenia (Grades III-IV).

*(3) Thrombocytopenia*. Data were available from 8 RCTs [10–13, 16, 17, 21–23, 31, 32]. These studies included 3298 patients. The pooled results showed statistically significant increases in the frequency of Grades III-IV thrombocytopenia with the use of bortezomib- and lenalidomide-based regimens compared with controls. The pooled RRs for thrombocytopenia were 1.54 (95% CI 1.07–2.22; *P* = 0.02) and 2.91 (95% CI 1.97–4.28; *P* < 0.00001), respectively. The pooled RR for all RCTs was 1.93 (95% CI 1.30–2.87; *P* = 0.001). There was heterogeneity among included RCTs (*I* ^2^ = 66%; *P* = 0.004) (Figure 7).

**Figure 8:**
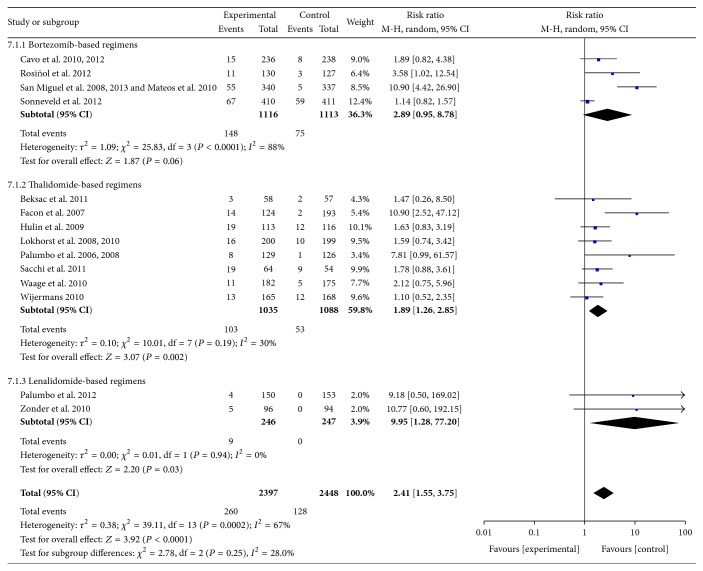
Comparison of novel agent-based regimens versus controls for gastrointestinal adverse events (Grades III-IV).

*(4) GI Events.* Data were available from 14 RCTs [10–12, 14–17, 19–24, 27–32]. These studies included 4845 patients. The most common GI AEs included nausea, diarrhea, constipation, and vomiting. Different authors have used various methods to assess GI AEs. In the present meta-analysis, the overall numbers of patients with Grades III-IV GI AEs were used. When this number was not available, all GI AEs were pooled together. The pooled results showed significant increases in the frequency of GI AEs with the use of novel agent-based regimens compared with controls, especially in the thalidomide- and lenalidomide-based regimens, but not in the bortezomib-based regimens. The pooled RR for all RCTs was 2.41 (95% CI 1.55–3.75; *P* < 0.0001). There was no heterogeneity among subgroups (*I* ^2^ = 28%; *P* = 0.25) (Figure 8).

**Figure 9:**
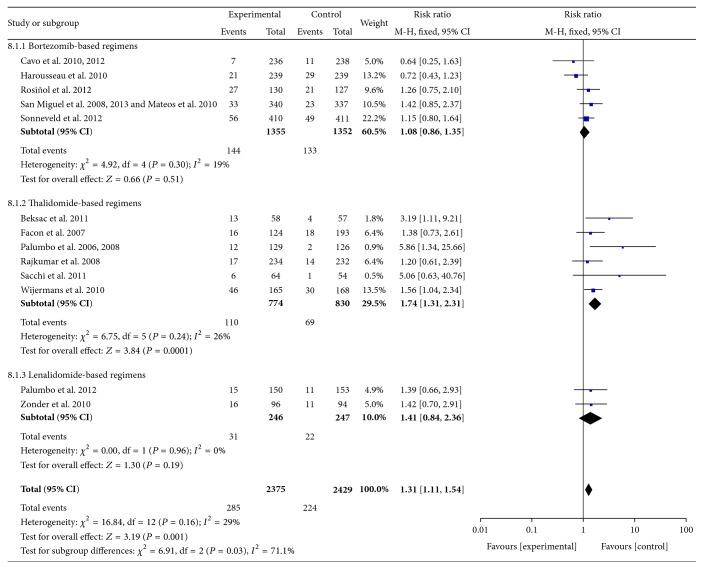
Comparison of novel agent-based regimens versus controls for infections (Grades III-IV).

*(5) Infections.* Data were available from 13 RCTs [10–18, 20–23, 31, 32]. These studies included 4804 patients. The overall number of patients with Grades III-IV infection symptoms (including pneumonia and herpes zoster) was used. The pooled results showed significant increases in the frequency of Grades III-IV infections in thalidomide-based regimens compared with controls. The pooled RRs were 1.74 (95% CI 1.31–2.31; *P* = 0.0001) for thalidomide-based regimens and 1.31 (95% CI 1.11–1.54; *P* = 0.001) for all RCTs. In addition, there was heterogeneity among subgroups (*I* ^2^ = 71.1%; *P* = 0.03), but not among included RCTs (*I* ^2^ = 29%; *P* = 0.16) (Figure 9).

**Figure 10:**
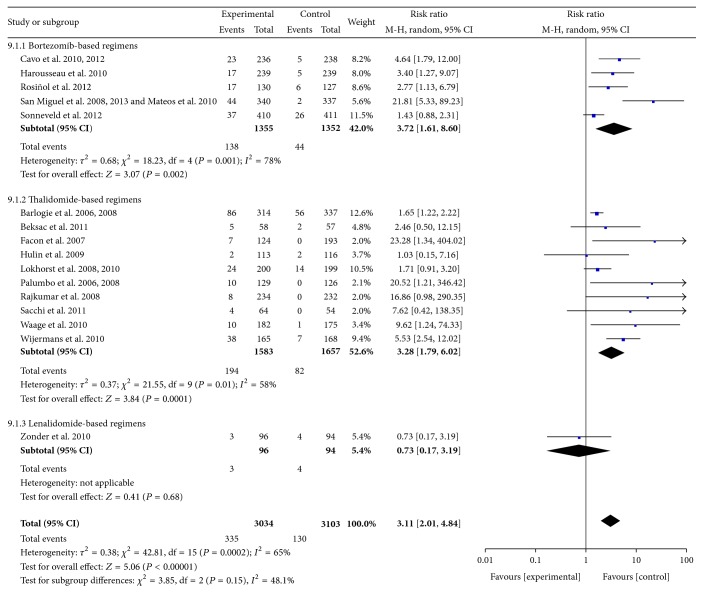
Comparison of novel agent-based regimens versus controls for peripheral neuropathy (Grades III-IV).

*(6) Peripheral Neuropathy (PN)*. Data were available from 16 RCTs [10–31]. These studies included 6137 patients. The pooled results showed significant increases in the frequency of Grades III-IV peripheral neuropathy symptoms with the use of bortezomib- and thalidomide-based regimens compared with controls. The pooled RRs were 3.72 (95% CI 1.61–8.6; *P* = 0.002) and 3.28 (95% CI 1.79–6.02; *P* = 0.0001), respectively. For all RCTs, the pooled RR was 3.11 (95% CI 2.01–4.84; *P* < 0.00001). There was no significant heterogeneity among subgroups (*I* ^2^ = 48.1%; *P* = 0.15) (Figure 10).

**Figure 11:**
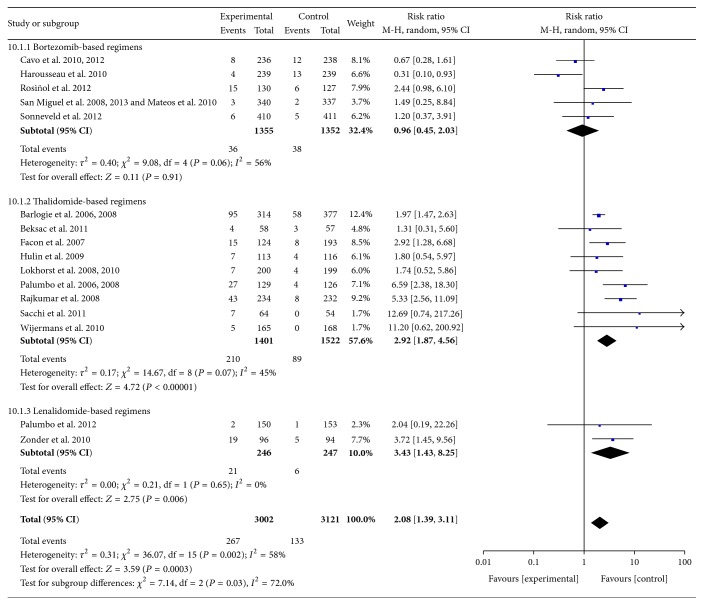
Comparison of novel agent-based regimens versus controls for thrombosis or embolism (Grades III-IV).

*(7) Thrombosis or Embolism*. Data were available from 16 RCTs [10–23, 25–32]. These studies included 6123 patients. The pooled results showed significant increases in the frequency of Grades III-IV thrombosis or embolism with the use of thalidomide- and lenalidomide-based regimens compared with controls. The pooled RRs were 2.67 (95% CI 1.87–4.56; *P* < 0.00001) and 3.43 (95% CI 1.43–8.25; *P* = 0.006), respectively. For all RCTs, the pooled RR was 2.08 (95% CI 1.39–3.11; *P* = 0.0003). There was significant heterogeneity among subgroups (*I* ^2^ = 72%; *P* = 0.03) (Figure 11).

### 3.6. Publication Bias

**Figure 12:**
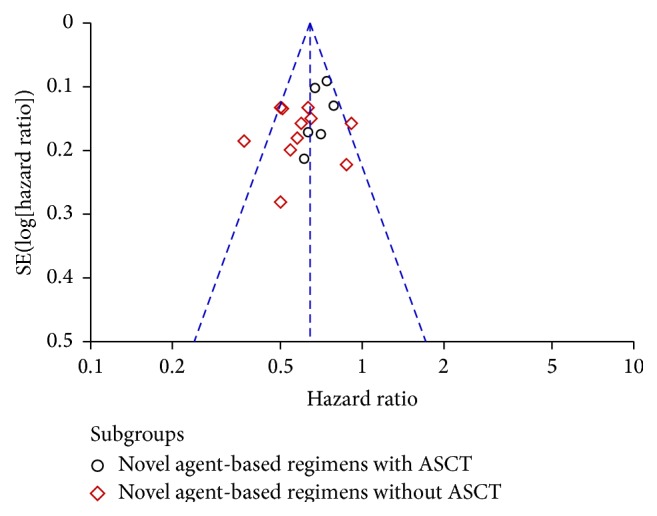
Funnel plot analysis of potential publication bias.

The funnel plot analysis was performed to address the potential publication bias of studies. The shapes of the funnel plots did not show any evidence of obvious asymmetry when taking all studies together (Figure 12) or when considering ASCT and no ASCT independently (figures not shown).

## 4. Discussion

Since the introduction of novel agents like IMiDs and bortezomib in the treatment of MM, there has been a significant improvement in survival and quality of life for patients with MM [6]. Bortezomib exerts its potent antimyeloma activity by inhibiting the survival of myeloma cell and restricting the development of tumor-associated blood vessels. IMiDs possess antiangiogenic and direct antitumor properties [33].

Several studies showed significant advantages of using novel agent-based regimens in patients with MM. Sonneveld et al. [34] observed that there are significant improvements in response and PFS/OS in patients with newly diagnosed MM (*n* = 1572) treated with bortezomib-based induction compared with non-bortezomib-based induction and that bortezomib was generally well tolerated. Nooka et al. [35] and Zeng et al. [36] demonstrated that bortezomib-based induction regimens offered significant clinical benefits in terms of CR, PFS, TTP, and OS, without increasing treatment-related mortality. The findings from Yang et al. [37] indicated that lenalidomide therapy significantly improved response rates and increased PFS in patients with newly diagnosed MM and in those who received previous antimyeloma therapy. Study from Zou et al. [38] suggested that there was a statistically significant difference for the outcome of PFS and OS favoring bortezomib arms versus controls. In addition, there was a statistically significant difference with lenalidomide arms versus controls for PFS but not OS. Fayers et al. [39] achieved an improvement of OS and PFS in previously untreated elderly patients with MM when thalidomide was added to MP, extending the median survival time by on average 20%.

In the present meta-analysis of efficacy, the pooled data suggested that novel agent-based regimens used in patients with MM induced benefits, which can be translated into higher CR and longer PFS and OS. Compared with non-novel agent-based induction regimens, the results of the present study demonstrated that induction therapy based on these novel agents resulted in significant improvements in CR and that this improvement was consistent across the individual studies that were analyzed. Results also showed that PFS was also significantly improved with bortezomib-based regimen compared with non-bortezomib-based regimens with or without ASCT. PFS was improved using lenalidomide-based regimens without ASCT. Compared with non-bortezomib-based induction, a strong trend toward improved OS was observed with bortezomib-based induction. Similar results could be seen in the subgroup of thalidomide-based regimens without ASCT, but they did not reach statistical significance in thalidomide-based regimens with ASCT or lenalidomide-based regimens without ASCT, which might be attributed to the small sample size of included studies in these two subgroups and short follow-up periods [25–28, 31, 32].

In the safety analysis, it was not possible to perform a summary statistic of all AEs because their definitions were different across trials. The most frequently reported AEs were mainly Grades III-IV. Based on the analysis of pooled data, hematological adverse events such as neutropenia, anemia, and thrombocytopenia were frequently reported in lenalidomide-based regimens. Bortezomib- and lenalidomide-based groups resulted in thrombocytopenia more often than in the control groups. As for the nonhematological AEs, it is not surprising that PN was the most common AE associated with bortezomib. A recent study from Tacchetti et al. [40] compared TD with VTD focusing on the incidence of PN showing that patients using VTD regimen had a higher incidence of PN in the induction phase which, however, was reversible and did not affect either their clinical outcomes or their ability to receive ASCT. Gene expression profiles (GEP) results showed that deregulated expression of genes involved in the cytoskeleton rearrangement and nervous system development and function may lead to the VTD-induced PN. Additionally, thalidomide was frequently associated with GI events, pneumonia, peripheral neuropathy, and thrombosis or embolism. Fatigue, diarrhea, and thrombosis could be seen in the lenalidomide group. Bagratuni et al. [41] argued that lenalidomide might be associated with a significant risk of venous thromboembolism, which was consistent with the present study. Most AEs could be improved or resolved by means of prompt modification or suspension of the agent dose [10–32]. In addition, some studies have shown that using lenalidomide resulted in a small increase in the risk of secondary primary tumor in both the first-line and maintenance settings.

Recently, a meta-analysis has shown that the use of lenalidomide in patients newly diagnosed with MM increased the risk of a secondary hematological cancer; this observation was mainly due to the combination of lenalidomide with melphalan [42]. Furthermore, it has been shown that lenalidomide increased the cumulative incidence of a second primary cancer compared with placebo [43]. A study from Attal et al. [44] suggested that an increased incidence rate of second primary cancers was observed in the lenalidomide group compared with the control group. In the present meta-analysis, Palumbo et al. [32] showed that the 3-year risk of a second primary tumor was 7% with MPR-R group and 3% with MP group. However, study from Zonder et al. [31] did not show similar results, which may be due to the small number of included articles.

The approach used in the present analysis has potential limitations that are common to all meta-analyses: inclusion of trials with different methodologies, different study designs, inconsistent endpoints, and different durations of follow-up. Given these differences among RCTs, some degree of statistical heterogeneity was anticipated. Heterogeneity between subgroups in the different novel agent-based regimens with or without ASCT could be seen with regard to PFS. However, there is little direct comparison between bortezomib, thalidomide, and lenalidomide, and it is difficult to confirm the superiority of one agent over the other. Recently, in a large randomized trial, the first (Intergroupe Francophone du Myélome 07-01, MM-020) trial, lenalidomide plus low-dose dexamethasone (Rd) for 18 cycles, showed no obvious advantage compared with MPT. However, continuous Rd has shown a significant improvement compared with MPT, with respect to PFS and OS [45].

A retrospective study of 411 patients reported that, compared with thalidomide and dexamethasone, patients receiving lenalidomide combined with dexamethasone achieved a longer time to progression and improved PFS and OS [46]. The results of the E1A06 trial were published in 2014 by the European Hematology Association and showed that there was no significant difference in treatment response or PFS or OS between MPR-R and MPT-T, which indicated that lenalidomide was not superior to thalidomide [47]. More clinical trials are needed to be conducted to address this issue. In addition, in the test for subgroups differences between novel agent-based regimens with ASCT and without ASCT, there was a significant difference with regard to PFS, but not in CR or OS, indicating that ASCT may not affect the comparison of the results in the present study [10–30]. A retrospective study of 318 elderly patients with newly diagnosed MM revealed that those treated with conventional chemotherapy (*n* = 192) achieved a median PFS of 19.1 months and a 5-year OS of 40%, while those receiving novel agent-based regimens (*n* = 88) achieved 24.5 months and 62%, those receiving conventional chemotherapy plus auto-SCT (*n* = 21) achieved 26.8 months and 63%, and those receiving novel agents plus auto-SCT (*n* = 17) achieved 35.2 months and 87% [48]. These results may indicate that novel agents may play a role that is as important as transplantation in the treatment of MM. An analysis from the International Myeloma Working Group consensus showed that novel agent-based induction regimens followed by autotransplantation achieved better responses resulting in extended PFS and even extended OS in patients with MM [49]. Further analysis could be focused on patients who underwent ASCT versus no ASCT based on the use of novel agents to figure out whether ASCT could be replaced by the regular use of novel agents including bortezomib, thalidomide, and lenalidomide. In addition, we presumed that different therapies in the maintenance or post-ASCT maintenance periods might be a potential cause of the total heterogeneity with regard to PFS and OS.

Stewart et al. [50] conducted a randomized phase 3 trial showing that thalidomide and prednisone maintenance after transplantation in patients with MM improves PFS but not OS. A study conducted by Palumbo et al. [32] also showed that the response rates and PFS benefit were noted in MM patients with MPR-R group compared to those with MPR group. A phase III, multicenter, randomized study compared the four-drug combination VMPT (bortezomib-melphalan-prednisone-thalidomide) followed by VT maintenance with VMP. The former showed higher response rate and longer PFS and OS [51].

Notably, the funnel plot analysis was performed to address the potential publication bias and confirmed that the results of the present study were reliable when taking all studies together or when considering ASCT and no ASCT independently. However, the limitations of this meta-analysis should be also taken into account. First, there were methodological problems in all the included trials. Most trials were not blinded. The allocation concealment was not used or unclear. Therefore, potential biases such as assessment bias and participant selection bias were likely to be present. Second, some of the analyses were based on published summary results instead of individual patient data, which are usually considered to be more reliable. Third, despite an exhaustive and thorough search, it is possible that negative RCTs results may not have been published.

## 5. Conclusions

Despite the AEs of novel agents in the present meta-analysis, there were clear advantages in terms of benefits and safety in the treatment of patients with MM using novel agent-based regimens like bortezomib, thalidomide, and lenalidomide, as previously recommended [52]. Novel agent-based therapy should be considered as promising induction regimens for patients with previously untreated MM. However, potential risk of AEs should be taken into account. Nevertheless, more information needs to be documented in extensive RCTs with different combinations of ASCT, novel agents, and traditional chemotherapy in both newly diagnosed and relapsing/refractory MM.

## Figures and Tables

**Table 1:** Characteristics of the included trials.

Trial	Author and year	Country	Number	Regimens	Follow-up (month)	Randomization	Blind	Allocation concealment	Withdrawal and dropout	ITT	Jadad score
VISTA NCT00111319	San Miguel et al. 2008, 2010, 2013 [10–12]	Europe, America, Asia	682	E: VMP *∗* 9 cyc C: MP *∗* 9 cyc	60	Yes	No	Unclear	Yes	Yes	3

IFM2005-01 NCT00200681	Harousseau et al. 2010 [13]	France, Belgium, Switzerland	482	E: VAD *∗* 4 cyc ± DCEP + ASCT C: BD *∗* 4 cyc ± DCEP + ASCT	32.2	Yes	No	Unclear	Yes	Yes	3

MM-BO2005 NCT01134484	Cavo et al. 2010, 2012 [14, 15]	Italy	474	E: VTD *∗* 3 cyc + ASCT + VTD *∗* 3 cyc C: TD *∗* 3 cyc + ASCT + TD *∗* 3 cyc	36	Yes	No	Yes	Yes	Yes	4

PETHEMA/GEM NCT00461747	Rosiñol et al. 2012 [16]	Spain	257	E: VTD *∗* 6 cyc + ASCT + T C: TD *∗* 6 cyc + ASCT + T	56.2	Yes	No	Unclear	Yes	Yes	3

HOVON-65/GMMG-HD4 ISRCTN:64455289	Sonneveld et al. 2012 [17]	Germany, Netherlands, Belgium	827	E: PAD *∗* 3 cyc + ASCT + P C: VAD *∗* 3 cyc + ASCT + T	41	Yes	No	No	Yes	Yes	3

THAL-MM-003 NCT00057564	Rajkumar et al. 2008 [18]	Australia, Spain, America	570	E: TD C: placebo + D (until progression)	22.6	Yes	Yes	Yes	Yes	Yes	5

IFM01/01 NCT00644306	Hulin et al. 2009 [19]	Belgium	229	E: MPT *∗* 12 cyc C: MP *∗* 12 cyc	47.5	Yes	No	Unclear	Yes	Yes	3

HOVON49 ISRCTN:90692740	Wijermans et al. 2010 [20]	Netherlands	333	E: MPT *∗* 8 cyc C: MP *∗* 8 cyc	48	Yes	No	Unclear	Yes	Yes	3

GISMM2001-A NCT00232934	Palumbo et al. 2006, 2008 [21, 22]	Italy	255	E: MP *∗* 6 cyc + T C: MP *∗* 6 cyc	38.4	Yes	No	Yes	Yes	Yes	5

IFM99-06 NCT00367185	Facon et al. 2007 [23]	France, Belgium, Switzerland	321	E: MPT *∗* 12 cyc C: MP *∗* 12 cyc	51.5	Yes	No	Unclear	Yes	Yes	3

NMSG#12 NCT00218855	Waage et al. 2010 [24]	Norway, Sweden, Denmark	357	E: MPT C: MP (until plateau phase)	42	Yes	Yes	Unclear	Yes	Yes	5

UARK98-026 NCT00083551	Barlogie et al. 2006, 2008 [25, 26]	America	668	E: total therapy 2 + T C: total therapy 2	96	Yes	No	Unclear	Yes	Yes	3

HOVON-5-/GMMG-HD3 ISRCTN:06413384	Lokhorst et al. 2008, 2010 [27, 28]	Netherlands, Germany, Belgium	556	E: VAD C: TAD	52	Yes	No	Unclear	Yes	Yes	3

TMSG-2005-001 NCT00934154	Beksac et al. 2011 [29]	Turkey	115	E: MPT *∗* 8 cyc C: MP *∗* 8 cyc	23	Yes	No	Unclear	No	Yes	2

MM03 NCT01274403	Sacchi et al. 2011 [30]	Italy	118	E: MPT *∗* 6–12 cyc C: MP *∗* 6–12 cyc	30	Yes	No	Unclear	Yes	Yes	3

SO232 NCT00064038	Zonder et al. 2010 [31]	America	192	E: LEX + DEX C: placebo + DEX (until progression)	47.2	Yes	Yes	Unclear	Yes	Yes	5

MM-015 NCT00405756	Palumbo et al. 2012 [32]	Europe, Australia, Israel	306	E: MPR *∗* 9 cyc + R C: (placebo + MP) *∗* 9 cyc	30	Yes	Yes	Unclear	Yes	Yes	5

E: experiment arm; C: control arm; cyc: cycles; VMP: bortezomib, melphalan; MP: melphalan, prednisolone; VAD: vincristine, adriamycin, and dexamethasone; prednisolone; DCEP: dexamethasone, cyclophosphamide, etoposide, and cisplatin; BD: bortezomib, dexamethasone; VTD: bortezomib, thalidomide, and dexamethasone; TD: thalidomide, dexamethasone; PAD: bortezomib, adriamycin, and dexamethasone; MPT: melphalan, prednisolone, and thalidomide; R: lenalidomide.

**Table 2:** Comparison of novel agent-based regimens with ASCT versus without ASCT.

	Study	CR (RR (95% CI))	PFS (HR (95% CI))	OS (HR (95% CI))
With ASCT	Barlogie et al. 2006, 2008 [25, 26]	1.44 [1.24, 1.67]	0.70 [0.50, 0.99]	0.70 [0.50, 0.99]
	Cavo et al. 2010, 2012 [14, 15]	4.03 [2.14, 7.62]	0.63 [0.45, 0.88]	0.63 [0.45, 0.88]
	Harousseau et al. 2010 [13]	4.37 [1.26, 15.14]	0.79 [0.61, 1.01]	0.79 [0.61, 1.01]
	Lokhorst et al. 2008, 2010 [27, 28]	1.50 [0.54, 4.16]	0.67 [0.55, 0.82]	0.67 [0.55, 0.82]
	Rosiñol et al. 2012 [16]	2.44 [1.50, 3.98]	0.61 [0.40, 0.93]	0.61 [0.40, 0.93]
	Sonneveld et al. 2012 [17]	4.15 [1.84, 9.37]	0.74 [0.62, 0.89]	0.74 [0.62, 0.89]
	Subtotal	2.54 [1.53, 4.23]	0.71 [0.64, 0.78]	0.71 [0.64, 0.78]
	Subgroup	*P* = 0.0003	*P* < 0.00001	*P* < 0.00001

Without ASCT	Beksac et al. 2011 [29]	1.02 [0.31, 3.33]	0.88 [0.57, 1.35]	0.88 [0.57, 1.35]
	Facon et al. 2007 [23]	5.50 [1.78, 16.97]	0.51 [0.39, 0.66]	0.51 [0.39, 0.66]
	Hulin et al. 2009 [19]	7.33 [0.92, 58.56]	0.60 [0.44, 0.81]	0.60 [0.44, 0.81]
	Palumbo et al. 2006, 2008 [21, 22]	6.51 [1.98, 21.37]	0.63 [0.48, 0.82]	0.63 [0.48, 0.82]
	Palumbo et al. 2012 [32]	3.04 [1.13, 8.16]	0.37 [0.26, 0.53]	0.37 [0.26, 0.53]
	Rajkumar et al. 2008 [18]	3.00 [1.21, 7.42]	0.50 [0.39, 0.65]	0.50 [0.39, 0.65]
	Sacchi et al. 2011 [30]	2.74 [0.95, 7.92]	0.50 [0.29, 0.87]	0.50 [0.29, 0.87]
	San Miguel et al. 2008, 2010, 2013 [10–12]	8.35 [4.68, 14.90]	0.55 [0.37, 0.81]	0.55 [0.37, 0.81]
	Waage et al. 2010 [24]	3.16 [1.39, 7.17]	0.91 [0.67, 1.25]	0.91 [0.67, 1.25]
	Wijermans et al. 2010 [20]	2.98 [1.65, 5.38]	0.65 [0.49, 0.87]	0.65 [0.49, 0.87]
	Zonder et al. 2010 [31]	6.12 [2.21, 16.92]	0.58 [0.40, 0.82]	0.58 [0.40, 0.82]
	Subtotal	3.91 [2.72, 5.60]	0.59 [0.53, 0.65]	0.59 [0.53, 0.65]
	Subgroup	*P* < 0.00001	*P* < 0.00001	*P* < 0.00001

Test for subgroup differences		*χ* ^2^ = 1.82, (*P* = 0.18), *I* ^2^ = 45.1%	*χ* ^2^ = 6.51, (*P* = 0.01), *I* ^2^ = 84.6%	*χ* ^2^ = 1.06, (*P* = 0.30), *I* ^2^ = 5.5%

## References

[1] Zou Y., Sheng Z., Lu H., Yu J. (2013). Continuous treatment with new agents for newly diagnosed multiple myeloma. *Anti-Cancer Drugs*.

[2] Kyle R. A., Gertz M. A., Witzig T. E. (2003). Review of 1027 patients with newly diagnosed multiple myeloma. *Mayo Clinic Proceedings*.

[3] Kohler B. A., Ward E., McCarthy B. J. (2011). Annual report to the nation on the status of cancer, 1975–2007, featuring tumors of the brain and other nervous system. *Journal of the National Cancer Institute*.

[4] Schaar C. G., le Cessie S., Snijder S. (2009). Long-term follow-up of a population based cohort with monoclonal proteinaemia. *British Journal of Haematology*.

[5] Landgren O., Rajkumar S. V., Pfeiffer R. M. (2010). Obesity is associated with an increased risk of monoclonal gammopathy of undetermined significance among black and white women. *Blood*.

[6] Kumar S. K., Rajkumar S. V., Dispenzieri A. (2008). Improved survival in multiple myeloma and the impact of novel therapies. *Blood*.

[7] Jadad A. R., Moore R. A., Carroll D. (1996). Assessing the quality of reports of randomized clinical trials: is blinding necessary?. *Controlled Clinical Trials*.

[8] Blade J., Samson D., Reece D. (1998). Criteria for evaluating disease response and progression in patients with multiple myeloma treated by high-dose therapy and haemopoietic stem cell transplantation. *British Journal of Haematology*.

[9] Tierney J. F., Stewart L. A., Ghersi D., Burdett S., Sydes M. R. (2007). Practical methods for incorporating summary time-to-event data into meta-analysis. *Trials*.

[10] San Miguel J. F., Schlag R., Khuageva N. K. (2008). Bortezomib plus melphalan and prednisone for initial treatment of multiple myeloma. *The New England Journal of Medicine*.

[11] Mateos M.-V., Richardson P. G., Schlag R. (2010). Bortezomib plus melphalan and prednisone compared with melphalan and prednisone in previously untreated multiple myeloma: updated follow-up and impact of subsequent therapy in the phase III VISTA trial. *Journal of Clinical Oncology*.

[12] San Miguel J. F., Schlag R., Khuageva N. K. (2013). Persistent overall survival benefit and no increased risk of second malignancies with bortezomib-melphalan-prednisone versus melphalan-prednisone in patients with previously untreated multiple myeloma. *Journal of Clinical Oncology*.

[13] Harousseau J.-L., Attal M., Avet-Loiseau H. (2010). Bortezomib plus dexamethasone is superior to vincristine plus doxorubicin plus dexamethasone as induction treatment prior to autologous stem-cell transplantation in newly diagnosed multiple myeloma: results of the IFM 2005-01 phase III trial. *Journal of Clinical Oncology*.

[14] Cavo M., Tacchetti P., Patriarca F. (2010). Bortezomib with thalidomide plus dexamethasone compared with thalidomide plus dexamethasone as induction therapy before, and consolidation therapy after, double autologous stem-cell transplantation in newly diagnosed multiple myeloma: a randomised phase 3 study. *The Lancet*.

[15] Cavo M., Pantani L., Petrucci M. T. (2012). Bortezomib-thalidomide-dexamethasone is superior to thalidomide-dexamethasone as consolidation therapy after autologous hematopoietic stem cell transplantation in patients with newly diagnosed multiple myeloma. *Blood*.

[16] Rosiñol L., Oriol A., Teruel A. I. (2012). Superiority of bortezomib, thalidomide, and dexamethasone (VTD) as induction pretransplantation therapy in multiple myeloma: a randomized phase 3 PETHEMA/GEM study. *Blood*.

[17] Sonneveld P., Schmidt-Wolf I. G. H., van der Holt B. (2012). Bortezomib induction and maintenance treatment in patients with newly diagnosed multiple myeloma: results of the randomized phase III HOVON-65/ GMMG-HD4 trial. *Journal of Clinical Oncology*.

[18] Rajkumar S. V., Rosiñol L., Hussein M. (2008). Multicenter, randomized, double-blind, placebo-controlled study of thalidomide plus dexamethasone compared with dexamethasone as initial therapy for newly diagnosed multiple myeloma. *Journal of Clinical Oncology*.

[19] Hulin C., Facon T., Rodon P. (2009). Efficacy of melphalan and prednisone plus thalidomide in patients older than 75 years with newly diagnosed multiple myeloma: IFM 01/01 trial. *Journal of Clinical Oncology*.

[20] Wijermans P., Schaafsma M., Termorshuizen F. (2010). Phase III study of the value of thalidomide added to melphalan plus prednisone in elderly patients with newly diagnosed multiple myeloma: the HOVON 49 study. *Journal of Clinical Oncology*.

[21] Palumbo A., Bringhen S., Caravita T. (2006). Oral melphalan and prednisone chemotherapy plus thalidomide compared with melphalan and prednisone alone in elderly patients with multiple myeloma: randomised controlled trial. *The Lancet*.

[22] Palumbo A., Bringhen S., Liberati A. M. (2008). Oral melphalan, prednisone, and thalidomide in elderly patients with multiple myeloma: updated results of a randomized controlled trial. *Blood*.

[23] Facon T., Mary J. Y., Hulin C. (2007). Melphalan and prednisone plus thalidomide versus melphalan and prednisone alone or reduced-intensity autologous stem cell transplantation in elderly patients with multiple myeloma (IFM 99-06): a randomised trial. *The Lancet*.

[24] Waage A., Gimsing P., Fayers P. (2010). Melphalan and prednisone plus thalidomide or placebo in elderly patients with multiple myeloma. *Blood*.

[25] Barlogie B., Pineda-Roman M., van Rhee F. (2008). Thalidomide arm of total therapy 2 improves complete remission duration and survival in myeloma patients with metaphase cytogenetic abnormalities. *Blood*.

[26] Barlogie B., Tricot G., Anaissie E. (2006). Thalidomide and hematopoietic-cell transplantation for multiple myeloma. *The New England Journal of Medicine*.

[27] Lokhorst H. M., Schmidt-Wolf I., Sonneveld P. (2008). Thalidomide in induction treatment increases the very good partial response rate before and after high-dose therapy in previously untreated multiple myeloma. *Haematologica*.

[28] Lokhorst H. M., van der Holt B., Zweegman S. (2010). A randomized phase 3 study on the effect of thalidomide combined with adriamycin, dexamethasone, and high-dose melphalan, followed by thalidomide maintenance in patients with multiple myeloma. *Blood*.

[29] Beksac M., Haznedar R., Firatli-Tuglular T. (2011). Addition of thalidomide to oral melphalan/prednisone in patients with multiple myeloma not eligible for transplantation: results of a randomized trial from the Turkish Myeloma Study Group. *European Journal of Haematology*.

[30] Sacchi S., Marcheselli R., Lazzaro A. (2011). A randomized trial with melphalan and prednisone versus melphalan and prednisone plus thalidomide in newly diagnosed multiple myeloma patients not eligible for autologous stem cell transplant. *Leukemia & Lymphoma*.

[31] Zonder J. A., Crowley J., Hussein M. A. (2010). Lenalidomide and high-dose dexamethasone compared with dexamethasone as initial therapy for multiple myeloma: a randomized Southwest Oncology Group trial (S0232). *Blood*.

[32] Palumbo A., Hajek R., Delforge M. (2012). Continuous lenalidomide treatment for newly diagnosed multiple myeloma. *The New England Journal of Medicine*.

[33] Andhavarapu S., Roy V. (2013). Immunomodulatory drugs in multiple myeloma. *Expert Review of Hematology*.

[34] Sonneveld P., Goldschmidt H., Rosiñol L. (2013). Bortezomib-based versus nonbortezomib-based induction treatment before autologous stem-cell transplantation in patients with previously untreated multiple myeloma: a meta-analysis of phase III randomized, controlled trials. *Journal of Clinical Oncology*.

[35] Nooka A. K., Kaufman J. L., Behera M. (2013). Bortezomib-containing induction regimens in transplant-eligible myeloma patients: a meta-analysis of phase 3 randomized clinical trials. *Cancer*.

[36] Zeng Z., Lin J., Chen J. (2013). Bortezomib for patients with previously untreated multiple myeloma: a systematic review and meta-analysis of randomized controlled trials. *Annals of Hematology*.

[37] Yang B., Yu R.-L., Chi X.-H., Lu X.-C. (2013). Lenalidomide treatment for multiple myeloma: systematic review and meta-analysis of randomized controlled trials. *PLoS ONE*.

[38] Zou Y., Lin M., Sheng Z., Niu S. (2014). Bortezomib and lenalidomide as front-line therapy for multiple myeloma. *Leukemia & Lymphoma*.

[39] Fayers P. M., Palumbo A., Hulin C. (2011). Thalidomide for previously untreated elderly patients with multiple myeloma: meta-analysis of 1685 individual patient data from 6 randomized clinical trials. *Blood*.

[40] Tacchetti P., Terragna C., Galli M. (2014). Bortezomib- and thalidomide-induced peripheral neuropathy in multiple myeloma: clinical and molecular analyses of a phase 3 study. *American Journal of Hematology*.

[41] Bagratuni T., Kastritis E., Politou M. (2013). Clinical and genetic factors associated with venous thromboembolism in myeloma patients treated with lenalidomide-based regimens. *American Journal of Hematology*.

[42] Palumbo A., Bringhen S., Kumar S. K. (2014). Second primary malignancies with lenalidomide therapy for newly diagnosed myeloma: a meta-analysis of individual patient data. *The Lancet Oncology*.

[43] McCarthy P. L., Owzar K., Hofmeister C. C. (2012). Lenalidomide after stem-cell transplantation for multiple myeloma. *The New England Journal of Medicine*.

[44] Attal M., Lauwers-Cances V., Marit G. (2012). Lenalidomide maintenance after stem-cell transplantation for multiple myeloma. *The New England Journal of Medicine*.

[45] Benboubker L., Dimopoulos M. A., Dispenzieri A. (2014). Lenalidomide and dexamethasone in transplant-ineligible patients with myeloma. *The New England Journal of Medicine*.

[46] Gay F., Hayman S. R., Lacy M. Q. (2010). Lenalidomide plus dexamethasone versus thalidomide plus dexamethasone in newly diagnosed multiple myeloma: a comparative analysis of 411 patients. *Blood*.

[47] Stewart A. K., Jacobus S. J., Fonseca R. E1A06: a phase III trial comparing melphalan, prednisone, and thalidomide (MPT) versus melphalan, prednisone, and lenalidomide (MPR) in newly diagnosed multiple myeloma (MM).

[48] Ozaki S., Harada T., Saitoh T. (2014). Survival of multiple myeloma patients aged 65-70 years in the era of novel agents and autologous stem cell transplantation. A multicenter retrospective collaborative study of the Japanese Society of Myeloma and the European Myeloma Network. *Acta Haematologica*.

[49] Cavo M., Rajkumar S. V., Palumbo A. (2011). International myeloma working group consensus approach to the treatment of multiple myeloma patients who are candidates for autologous stem cell transplantation. *Blood*.

[50] Stewart A. K., Trudel S., Bahlis N. J. (2013). A randomized phase 3 trial of thalidomide and prednisone as maintenance therapy after ASCT in patients with MM with a quality-of-life assessment: the National Cancer Institute of Canada Clinicals Trials Group Myeloma 10 Trial. *Blood*.

[51] Palumbo A., Bringhen S., Larocca A. (2014). Bortezomib-melphalan-prednisone-thalidomide followed by maintenance with bortezomib-thalidomide compared with bortezomib-melphalan-prednisone for initial treatment of multiple myeloma: updated follow-up and improved survival. *Journal of Clinical Oncology*.

[52] Yaqub S., Ballester G., Ballester O. (2013). Frontline therapy for multiple myeloma: a concise review of the evidence based on randomized clinical trials. *Cancer Investigation*.

